# The Role of Thioredoxin in Mitigating Ammonia-Induced Oxidative Stress in Nile Tilapia (*Oreochromis niloticus*)

**DOI:** 10.3390/ani16111580

**Published:** 2026-05-22

**Authors:** Yu Yu, Yanghui Chen, Yingying Chang, Junliang Luo, Haoze Li, Jinyuan Feng, Minghui Zhu, Bei Wang, Yu Huang, Jichang Jian

**Affiliations:** Fisheries College, Guangdong Ocean University, Zhanjiang 524091, China; 18284852446@163.com (Y.Y.); chenyanghui1022@163.com (Y.C.); 17266909989@163.com (Y.C.); luojunliang1022@163.com (J.L.); 19929950228@163.com (H.L.); 19838139467@163.com (J.F.); 13378478183@163.com (M.Z.); wong19820204@126.com (B.W.)

**Keywords:** Nile tilapia, *TRX*, oxidative stress, ammonia exposure

## Abstract

Thioredoxin (TRX) is an important redox-related protein, which plays a key role in maintaining redox balance in cells. However, the role of TRX in ammonia exposure of *Oreochromis niloticus* is still unknown. In this study, we successfully cloned the *TRX* gene (OnTRX) of *O. niloticus* and performed systematic bioinformatics analysis. The results of multi-sequence alignment and phylogenetic tree analysis showed that On*TRX* is highly conserved in vertebrates, but exhibits low sequence similarity with *TRX* homologs from arthropods (crustaceans). The tissue distribution of On*TRX* and its transcriptional response to ammonia challenge were determined by qRT-PCR, and we further investigated the relationship between On*TRX* and the response of the Nile tilapia epithelial cell line (TSE-04) to NH_4_Cl treatment. The results showed that On*TRX* exhibited tissue-specific expression, with a relatively high expression level in gill tissue. Ammonia exposure could significantly induce the expression of On*TRX* in the gill, head kidney, intestine, skin, liver, and spleen of tilapia. In TSE-04 cells, On*TRX* overexpression was associated with reduced NH_4_Cl-induced morphological damage, a lower proportion of apoptotic cells, and altered transcript levels of several stress-related genes. Collectively, these findings indicate that On*TRX* is likely involved in the regulatory response of Nile tilapia to ammonia-induced stress, while the underlying molecular mechanisms thereof remain to be further elucidated.

## Simple Summary

Ammonia is a toxic waste product that accumulates in aquaculture water, impairing fish health and reducing farming efficiency. Nile tilapia is among the most widely farmed fish globally, yet the biological mechanisms underlying its response to ammonia stress remain incompletely understood. In this study, we investigated whether thioredoxin—a naturally occurring protective protein in the body—helps Nile tilapia counteract ammonia-induced damage. We found that ammonia exposure increased oxidative stress, caused tissue injury, and disrupted normal cellular homeostasis. Concurrently, thioredoxin levels underwent significant changes, indicating thioredoxin appears to play a key protective role during stress exposure. Our results suggest that thioredoxin mitigates oxidative damage and preserves normal cellular function when tilapia are challenged with ammonia. These findings enhance our understanding of how fish defend against poor water quality and may inform the development of improved health management strategies in aquaculture. Ultimately, this knowledge could support more sustainable fish farming practices and contribute to enhanced food production.

## 1. Introduction

Nile tilapia (*Oreochromis niloticus* L.) is one of the freshwater fish with important economic value [1]. Because of its good salt and alkali tolerance, fast growth, and good cold and drought resistance in different environments, it has been widely cultivated all over the world [2,3,4,5]. The increase in intensive farming density has caused the concentration of ammonia exposure in water to rise continuously, which has become the main environmental factor restricting the healthy development of tilapia industry. High ammonia exposure not only inhibits the growth of fish and reduces feed efficiency, but also destroys organ structures such as gill, liver, and kidney, increases pathogen susceptibility, and even leads to large-scale deaths in severe cases, bringing huge economic losses to farmers [6,7]. After ammonia exposure enters fish, it causes a series of biochemical reactions in gills and liver, leading to the outbreak of reactive oxygen species (ROS), breaking the redox balance of cells and inducing oxidative stress [8,9]. Long-term oxidative stress damages lipid, proteins, and nucleic acids, inhibits mitochondrial function, activates apoptotic pathways, and ultimately causes tissue necrosis and impaired immune function [10]. Many studies have shown that oxidative stress is a key factor contributing to fish damage induced by ammonia nitrogen toxicity. The thioredoxin system is widely present in various fish species and is recognized as an important endogenous redox regulator. However, the molecular characteristics of thioredoxin (TRX) in *O. niloticus*, as well as its specific role in coping with ammonia-induced stress, remain unclear. Specifically, it remains unknown whether the *TRX* in *O. niloticus* is involved in the response process to ammonia toxicity [11,12,13].

TRX is a small molecule redox protein, which reduces disulfide bonds in a NADPH-dependent manner through its conserved -Cys-Gly-Pro-Cys- active center, thus maintaining the reduction state of intracellular proteins [14]. TRX is not only the core downstream molecule of the Nrf2/ARE signaling pathway, but also can directly scavenge ROS and inhibit inflammatory cytokines [15,16,17]. Previous studies have shown that *TRX* is involved in antioxidant regulation and immune responses in several aquatic animals, including crustaceans and teleost fish [18,19]. However, the oxidative stress function of On*TRX* in Nile tilapia is still unknown.

Therefore, to promote the sustainable development of the Nile tilapia breeding industry, it is very important to deeply analyze its antioxidant system. In this study, TRX gene (On*TRX*) in Nile tilapia was successfully identified and characterized, and the biological function of the On*TRX* gene under ammonia stress was emphatically discussed. This study provides a new perspective for analyzing the toxic mechanism of ammonia exposure in Nile tilapia, and also provides an important theoretical basis for promoting healthy and sustainable aquaculture practice. Herein, we cloned On*TRX* from Nile tilapia, characterized its tissue expression pattern, and systematically explored its functional mechanisms in alleviating ammonia-induced oxidative stress and apoptosis.

## 2. Materials and Methods

### 2.1. Animal Preparation and Sample Collection

Healthy Nile tilapia (weight: 50 ± 5 g) was obtained from a local aquaculture farm in Zhanjiang, Guangdong, China Province. These fish swim normally, without obvious scars or lesions, and their fins and scales are intact. Before the experiment began, we disinfected all the water tanks, and then raised these fish in a laboratory environment for 14 days. Fish were maintained in a static water system rather than a flow-through system. During the 14-day acclimation period, the water was renewed once daily. During acclimation, ammonium, nitrite, nitrate, and chloride were monitored daily and kept within stable, safe ranges to ensure consistent rearing conditions. During the 72 h ammonia exposure period, no water exchange was performed; instead, NH_4_Cl was supplemented as needed to maintain the target ammonia concentration. The volume of their feeding box was 50 L, of which the volume of aquaculture water was 40 L. The water temperature should be kept at 27 ± 1 °C, the pH value should be in the range of 7.4–7.6, and the dissolved oxygen content should be in the range of 5.2–5.9 mg/L. For the ammonia exposure experiment, fish were randomly assigned to the control group and the NH_4_Cl treatment groups. Three fish were sampled at each time point (0, 6, 12, 24, 48, and 72 h), for a total of 18 fish. Tissue samples were collected from three individual tilapia, each regarded as an independent biological replicate.

### 2.2. Extraction of RNA and Synthesis of cDNA

Using an RNA extraction kit (TaKaRa, Dalian, China), we extracted total RNA from each collected sample, followed by cDNA synthesis via a reverse transcription kit (TaKaRa, Dalian, China). All operations were conducted according to our previous research [20].

### 2.3. Cloning and Bioinformatics Analysis of OnTRX

On*TRX* was cloned using the previous method [21]. Briefly, the primers were designed according to the sequences published in the NCBI database (XM_003447534.4) and the open reading frame (ORF) of On*TRX* was cloned from the cDNA of the above tissues. The primers used in this study (all designed by Primer 5.0 software) were listed in Table 1. After sequencing, the domain of On*TRX* was predicted by using the SMART tool. The phylogenetic tree was constructed by MEGA software (version 11.0) and NJ method, and the reliability was verified by 1000 self-expanding replicates. The secondary structure of amino acids of OnTRX was elucidated by network protein sequence analysis (NPS@), and its three-dimensional structure was constructed by SWISS-MODEL (http://swissmodel.expasy.org/).

### 2.4. Ammonia Exposure of Tilapia

Healthy tilapia was temporarily raised in an experimental water tank. After thorough disinfection and sterilization, the containers for feeding fish were divided into control and ammonia-exposed groups, with three replicate tanks per group and 10 fish in each tank. Ammonia stress was induced by dissolving NH_4_Cl in the culture water to achieve a target concentration of 5 mg/L total ammonia nitrogen [22]. Fish in the control group were maintained under the same conditions in ammonia-free water. Tissue samples (liver, gill, spleen, intestine, skin, and head kidney) were collected before ammonia exposure (0 h) and 3, 6, 12, 24, 48, and 72 h after exposure, respectively. For gill sampling, gill filament tissue was collected. For intestinal sampling, the middle section of the intestine was dissected, and the intestinal contents were removed before storage. During the 72 h exposure period, water quality parameters were monitored regularly, and the ammonia concentration was maintained at the target level by adjusting the exposure solution as needed. At each sampling time point, three fish were randomly collected from each group. All samples were flash-frozen in liquid nitrogen immediately after collection and stored at −80 °C for further analysis. Among them, the samples collected before exposure (0 h) were used as the control group.

### 2.5. Construction of Recombinant pCDNA3.1 Vector (pCDNA3.1-OnTRX)

In the present experiment, the recombinant vector was constructed with reference to the instructions of the Cloning Express Superspeed One-Step Cloning Kit V3 (Vazyme, Nanjing, China). To put it simply: firstly, using pCDNA3.1 as a template, the pCDNA3.1 reverse cloning primers were designed using Primer 5.0 software for PCR amplification, and the target vectors were linearized and recycled. The homologous arm (HA) sequence of the target vector was integrated into the primer for On*TRX*, and the target fragment was gel-recovered and purified after PCR amplification. Then, the above recycled products were proportionally connected at 50 °C for 15 min. The ligation products were transferred into DH5α (*Escherichia coli*), and positive clones were obtained after screening, and then sequenced and identified. After a large number of strains with correct sequence were amplified, the recombinant plasmid was isolated and purified by Endo-free plasmid Mini kit II (Vazyme, Nanjing, China) to meet the needs of subsequent experiments.

### 2.6. Revival of the TSE-04 Tilapia Skin Epithelial Cell Line and Recombinant Plasmid Transfection

TSE-04 (tilapia skin epithelial cell line) utilized in this work originated from our earlier research [23]. After resuscitation of TSE-04 cells, they were placed in cell culture flasks (Jet Biofil, Guangzhou, China) and cultured using L-15 medium (Gibco, Boston, MA, USA) containing 10% fetal bovine serum (FBS; Gibco, Boston, USA). Cells were maintained at 28 °C without CO_2_ supplementation, and no antibiotics were added to the culture medium. Once the cells were stabilized, they were seeded into 6-well plates (Jet Biofil, Guangzhou, China). When the cells grew adherently to the bottom of the plate and reached 80% density, the pCDNA3.1-On*TRX* plasmid was transfected as required in the instructions using Invitrogen Lipofectamine 2000 Transfection Reagent (Shanghai Cytoch Biotechnology Co., Ltd., Shanghai, China). After the transfection was completed, the culture medium was replaced in time according to the actual situation. Total RNA was extracted from TSE-04 48 h following transfection according to the methods detailed in 2.8 to verify the efficient overexpression of On*TRX* in the cells.

### 2.7. Ammonia Exposure Experiment of TSE-04

After it was confirmed that TSE-04 cells overexpressed On*TRX*, blank TSE-04 cells transfected with pCDNA3.1 empty vector and TSE-04 cells overexpressed On*TRX* were inoculated into new 6-well plates. When the cell density reached 90%, three groups were set up and treated accordingly: the blank group was blank TSE-04 cells without NH_4_Cl stimulation (control group); treated with NH_4_Cl with the final concentration of 74 mM [23,24], it was set as the pCDNA3.1 NH_4_Cl group; TSE-04 cells overexpressing On*TRX* were exposed to the final concentration of NH_4_Cl of 74 mM, which was set as the pCDNA3.1-On*TRX* NH_4_Cl group. TSE-04 before stimulation was collected as 0 h, and cell samples were collected at 3, 6, 12, 24, and 48 h after stimulation to extract total RNA. After 48 h of stimulation, the above three groups of TSE-04 cells were imaged using a Leica microscope (Leica, Wetzlar, Germany). Each group had 3 biological replicates.

### 2.8. Quantitative Real-Time PCR (qRT-PCR) Analysis

With the use of an RNA extraction kit (TaKaRa, Dalian, China), total RNA was isolated from each harvested sample, and subsequent cDNA synthesis was performed using a reverse transcription kit (TaKaRa, Dalian, China). All experimental operations were carried out in strict accordance with the instructions provided by the manufacturer.

qRT-PCR was performed using a 10 μL reaction system. The amplification program consisted of an initial denaturation step followed by 40 cycles of amplification. Primer specificity was confirmed by melt-curve analysis, and each primer pair produced a single peak. Primer amplification efficiencies were validated prior to analysis and were all greater than 90%. Each sample was analyzed with three technical replicates. *β-actin* was used as the reference gene for normalization, and the relative expression levels were determined using the 2^−ΔΔCt^ method.

### 2.9. Detection of Apoptosis by Flow Cytometry

According to the grouping described in Section 2.7, TSE-04 cells were inoculated on 6-well plates. When the confluence of cells exceeded 90%, the cells were stimulated by NH_4_Cl and grouped according to the scheme detailed in Section 2.7. After 24 h of stimulation, three groups of TSE-04 cells were stained using the Annexin V Alexa Fluor488/PI (CA1040, Solarbio, Beijing, China) and subsequently recorded by AttuneTMNxT flow cytometry (Invitrogen™, Thermo, 3AFC200040623, Shanghai, China).

### 2.10. Statistical Analysis and Graphical Representation of the Research Data

Data were expressed as mean ± standard deviation (SD). One-way analysis of variance or Student’s *t*-test was used for statistical analysis, depending on the specific method. Student’s *t*-test was used for comparisons between two groups, whereas one-way analysis of variance (ANOVA, SPSS Statistics 26) followed by Tukey’s multiple-comparison test was used for comparisons among three or more groups or among multiple time points. Before analysis, data were checked for normality and homogeneity of variance using the functions available in GraphPad Prism version 9.5. In figures with letter annotations, different letters indicate statistically significant differences among groups based on Tukey’s multiple-comparison test (*p* < 0.05). All statistical analyses were performed using GraphPad Prism version 9.5. All diagrams were created using Adobe Photoshop CC (San Jose, CA, USA).

## 3. Results

### 3.1. Sequence Analysis of TRX

**Figure 1:**
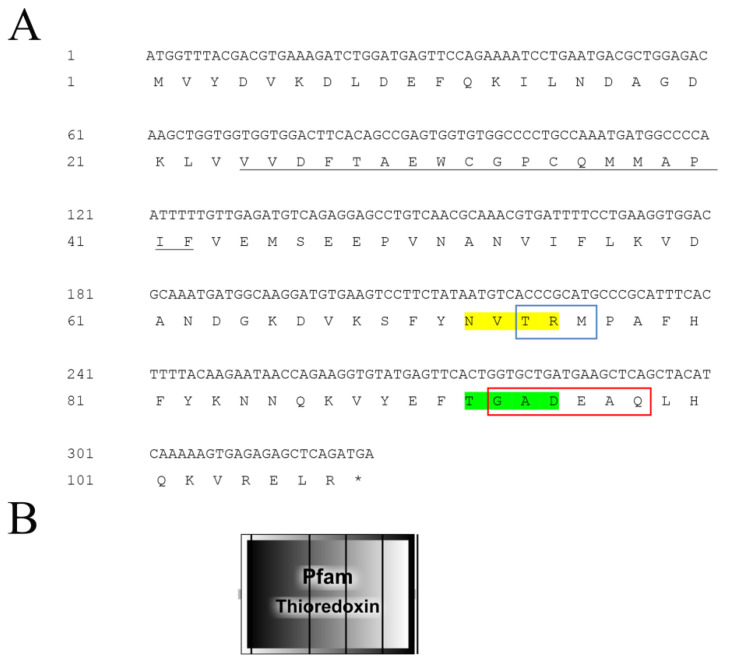
Sequence analysis and conserved domain prediction of thioredoxin (OnTRX) in Nile tilapia (*Oreochromis niloticus*). (**A**) Gene sequence of OnTRX in Nile tilapia (*Oreochromis niloticus*). *: terminator; yellow highlight: N-glycosylation site; green highlight: phosphorylation site of casein kinase II; red box: N- myristic acylation site; blue box: C- terminal localization signal site of microbody; black underline: active site of thioredoxin family. (**B**) Prediction of conserved domains of OnTRX in Nile tilapia (*Oreochromis niloticus*). The thioredoxin domain is marked in black.

**Figure 2:**
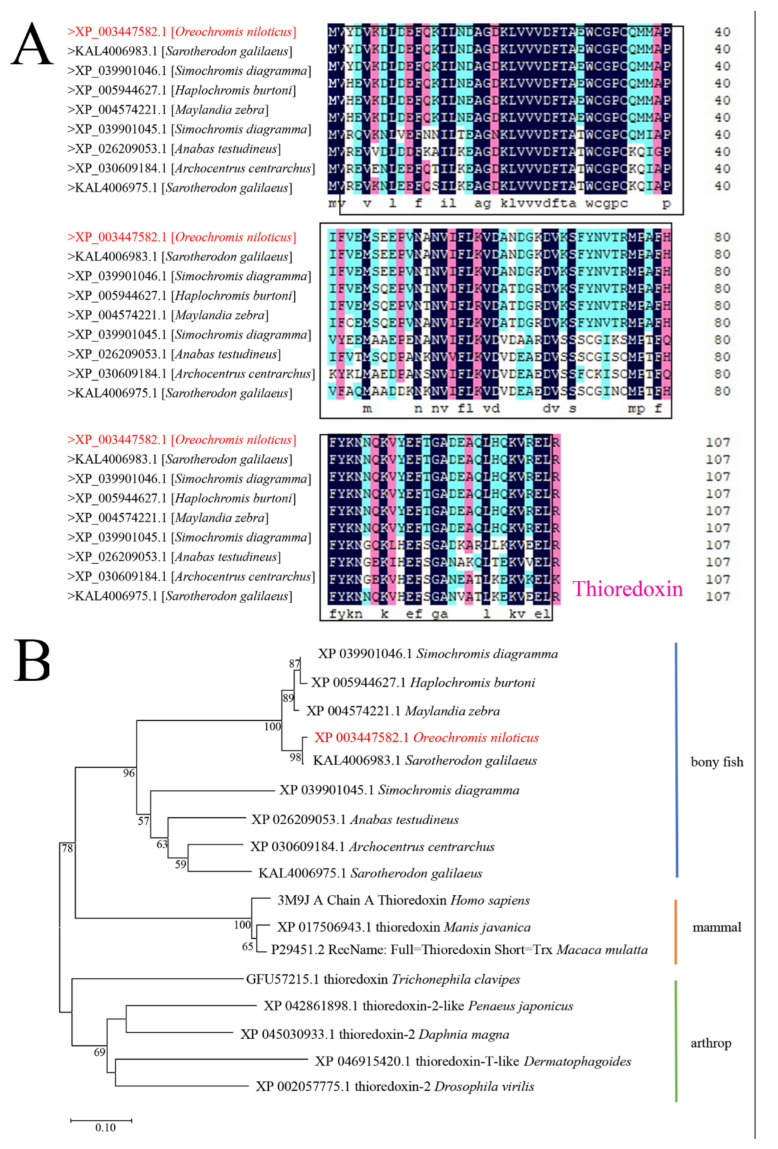
(**A**) Multi-sequence alignment of OnTRX in Nile tilapia (*Oreochromis niloticus*) and different species. (**B**) The phylogenetic tree of OnTRX from Nile tilapia (*Oreochromis niloticus*) and orthologs from other species was generated employing the NJ algorithm with MEGA 11, and OnTRX was marked in red for emphasis.

**Figure 3:**
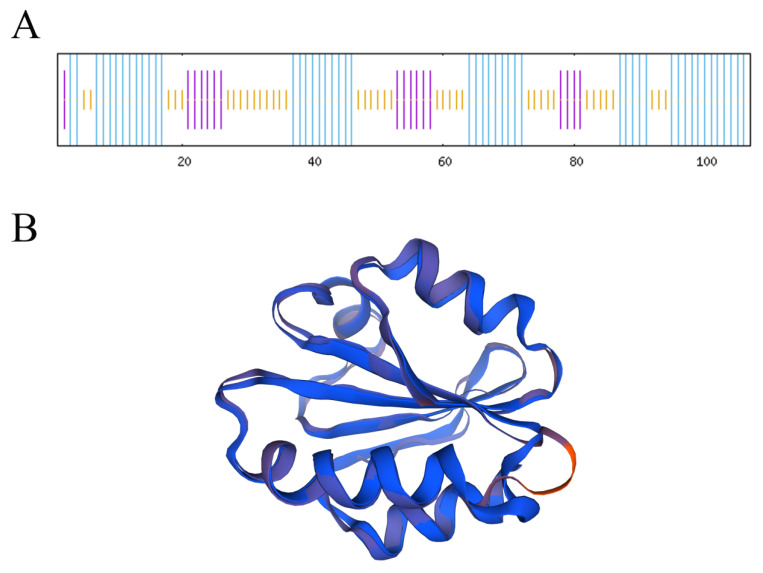
(**A**) Secondary structure of OnTRX in Nile tilapia (*Oreochromis niloticus*) predicted with NPS@. (**B**) Three-dimensional structural modeling of OnTRX in Nile tilapia (*Oreochromis niloticus*) by SWISS-MODEL.

The ORF of On*TRX* was 324 bp, encoding a protein composed of 107 amino acids (Figure 1A). The predicted structure of the SoftBerry-Psite program showed that the amino acid sequence contained one N-glycosylation site, one casein kinase II phosphorylation site, one N-myristoylation site, one micro-C- terminal localization signal site, and one thioredoxin family active site (Figure 1A). Structural prediction showed that OnTRX contains a thioredoxin domain, and thioredoxin is a small redox protein existing in all organisms. In the process of NADPH-dependent reduction, thioredoxin reductase (TR) catalyzes NADPH to reduce oxidized thioredoxin (Trx), and the reduced TRX further directly reduces disulfide in the substrate protein, thus exerting its redox function (Figure 1B). Through multi-sequence comparison, OnTRX among different species is highly conserved (Figure 2A). The phylogenetic tree showed that the OnTRX sequence of vertebrates is highly conserved in the evolution process. Compared with arthropods, mammals and teleost OnTRX are closer (Figure 2B). The secondary structure prediction using NPS@ showed that the sequence contains 46.73% α-helix, 16.82% extended chain, and 36.45% random curl (Figure 3A). The 3D model of On*TRX* is shown in Figure 3B. This structure presents On*TRX*’s “β-folded sandwich” model, which ensures its conformational flexibility and stability in the redox cycle.

### 3.2. Relative Expression Patterns of OnTRX Across Various Organs

**Figure 4:**
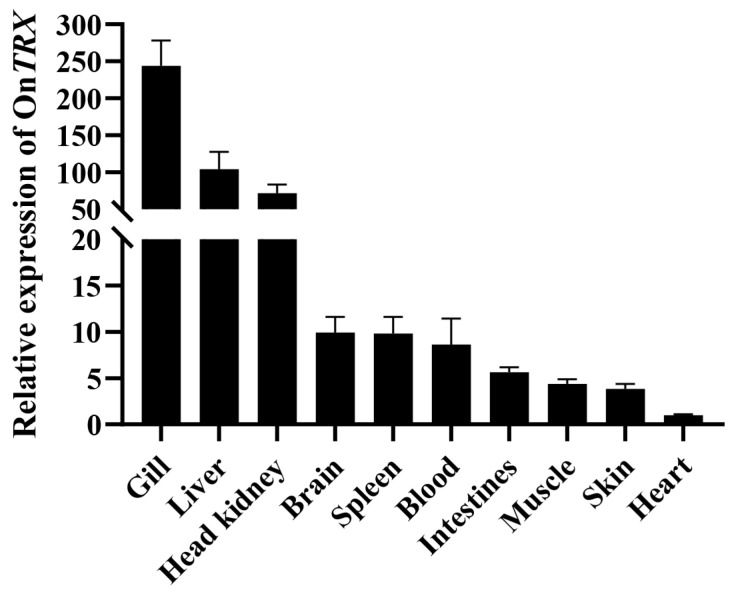
Relative expression of On*TRX* in different organs of Nile tilapia (*Oreochromis niloticus*). Relative expression levels were normalized to *β-actin* and calculated using the 2^−ΔΔCt^ method.

As shown in Figure 4, the histogram showed the relative expression levels of the On*TRX* in different tissues, with the highest expression level of the On*TRX* in the gill, then followed by the liver, head kidney, brain, spleen, blood, intestines, muscles, skin, and heart.

### 3.3. Temporal Expression Profile of OnTRX in Tilapia Tissues Subjected to NH_4_Cl Exposure

**Figure 5:**
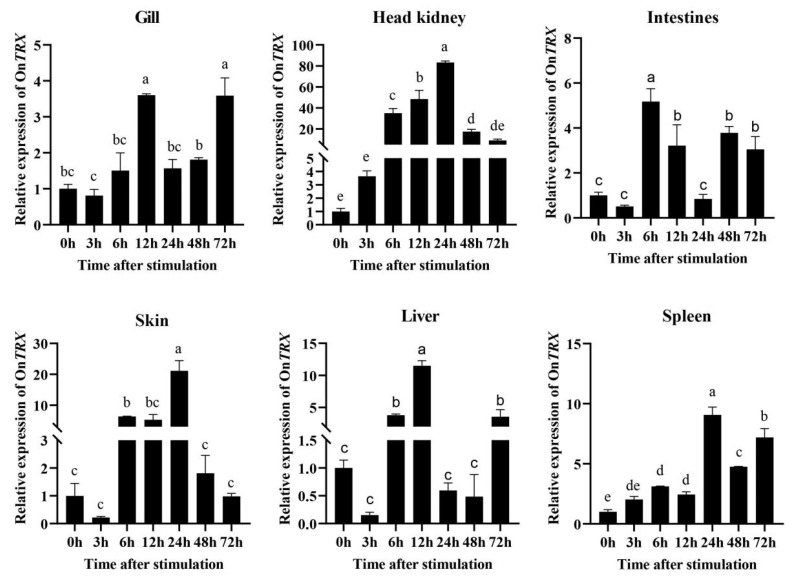
qRT-PCR analysis of relative On*TRX* expression in Nile tilapia (*Oreochromis niloticus*) tissues upon ammonia exposure. Note: Different letters indicate significant differences (*p* < 0.05).

As shown in Figure 5, NH_4_Cl could significantly induce the expression level of On*TRX* in the gill, head kidney, intestine, skin, liver, and spleen of Nile tilapia. In these tissues, the expression of On*TRX* in the head kidney, skin, and spleen reached the peak after 24 h of ammonia exposure, which was about 80 times, 18 times and 21 times of the 0 h level, respectively. During 72 h of ammonia exposure, the expression of On*TRX* in gill, intestine, and liver showed an upward trend at first, then decreased, and finally rose again.

### 3.4. Response of OnTRX to NH_4_Cl Exposure over Time in TSE-04 Cells

**Figure 6:**
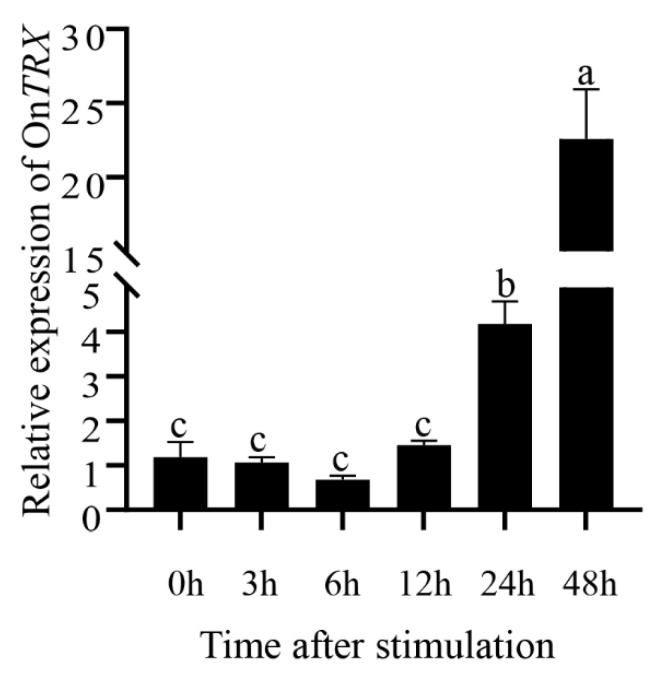
qRT-PCR assay of relative On*TRX* expression in TSE-04 cellsderived from Nile tilapia (*Oreochromis niloticus*) upon ammonia exposure. Note: Different letters indicate significant differences (*p* < 0.05).

As shown in Figure 6, the expression of On*TRX* in TSE-04 changed significantly after exposure to NH_4_Cl. Specifically, after NH_4_Cl stimulation, the relative expression of On*TRX* showed obvious time-dependent changes. At 0, 3, 6, and 12 h after stimulation, there was no significant difference in the relative expression level of On*TRX*; its expression began to increase gradually after 12 h, increased significantly at 24 h, and reached the peak at 48 h.

### 3.5. The Plasmid Transfection Efficiency Was Assessed via qPCR

**Figure 7:**
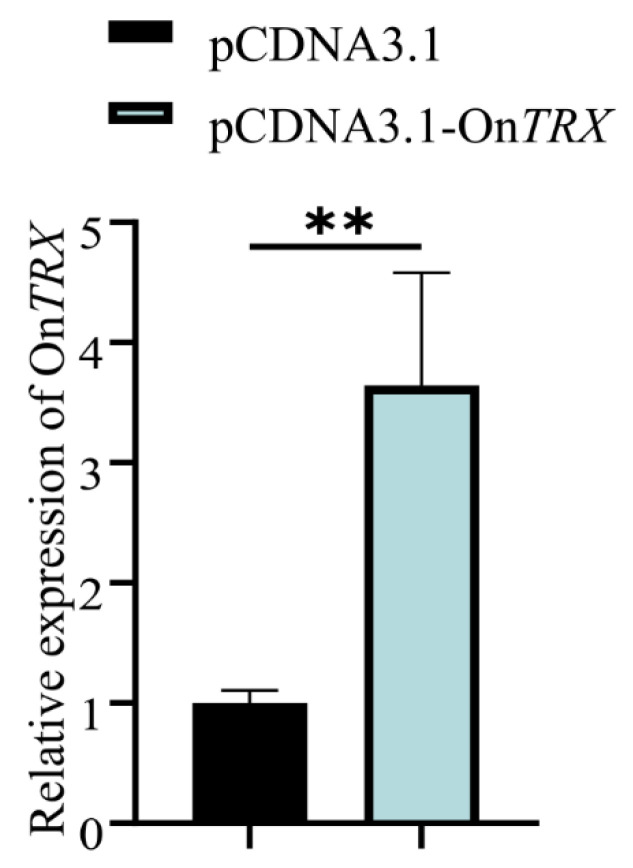
Relative On*TRX* expression in Nile tilapia (*Oreochromis niloticus*) of the two groups was quantified via qRT-PCR. Note: Asterisks indicate statistically significant differences between the two groups (*p* < 0.05), the same below.

qRT-PCR analysis showed that the relative mRNA expression level of On*TRX* in the pCDNA3.1-OnTRX group was significantly higher than that in the pCDNA3.1 group, indicating successful transcriptional overexpression of On*TRX* in TSE-04 cells (Figure 7).

### 3.6. Observation of NH_4_Cl-Treated TSE-04 Cells via Optical Microscopy

**Figure 8:**
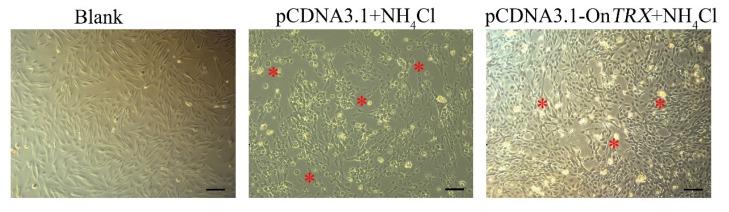
In comparison with the three TSE-04 cell groups (derived from Nile tilapia, *Oreochromis niloticus*) observed under an optical microscope (OM), red asterisks signify cellular atrophy. Scale bar = 100 μm.

Figure 8 shows the morphological changes of three groups of TSE-04 cells after NH_4_Cl treatment. Before adding NH_4_Cl, the cells were closely arranged, regular in shape and well connected, with no obvious signs of cell damage or death (Figure 8, blank). After NH_4_Cl treatment, apparent morphological changes were observed in TSE-04 cells compared with the blank group. The cells were swollen and deformed, and the edges became brighter, and the intercellular connection became loose (Figure 8, pCDNA3.1 + NH_4_Cl). In the pCDNA3.1-On*TRX* + NH_4_Cl group, cellular morphological damage was alleviated compared with that in the pCDNA3.1 + NH_4_Cl group. Although some cellular damage was still observed, the cells appeared relatively more regular in shape and showed comparatively closer intercellular connections (Figure 8, pCDNA3.1-On*TRX* + NH_4_Cl).

### 3.7. Effects of NH_4_Cl on TSE-04

**Figure 9:**
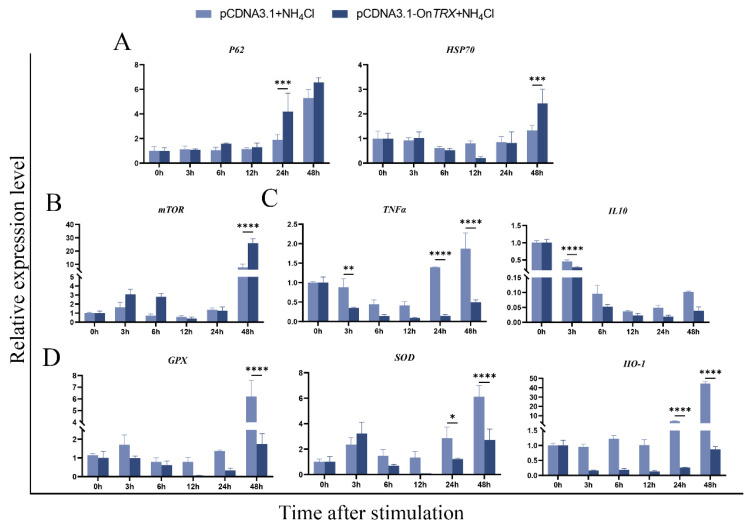
Relative expression levels of relevant genes in Nile tilapia (*Oreochromis niloticus*) following NH_4_Cl treatment. Note: (**A**) qRT-PCR was utilized to determine the expression abundance of autophagy-related gene (*HSP70*, *P62*); (**B**) cell growth and metabolic central pathway (*mTOR*); (**C**) inflammation and immunoregulatory factors (*TNFα*, *IL10*); (**D**) oxidative stress defense gene (*GPX*, *SOD*, *HO-1*). * *p* < 0.05, ** *p* < 0.01, *** *p* < 0.001, **** *p* < 0.0001.

We investigated whether On*TRX* overexpression was associated with altered stress responses in TSE-04 cells. Therefore, after the cells were exposed to NH_4_Cl, we examined the expression of genes related to cell growth and metabolism, inflammatory response, antioxidant defense, and autophagy. As shown in Figure 9B, the transcript level of *mTOR*, a gene associated with cell growth and metabolic regulation, increased significantly within 48 h after NH_4_Cl stimulation. At 48 h, the expression level of *mTOR* in the pCDNA3.1-OnTRX + NH_4_Cl group was about twice that in the pCDNA3.1 + NH_4_Cl group. As shown in Figure 9C, the transcript level of the pro-inflammatory factor *TNFα* in the pCDNA3.1-On*TRX* + NH_4_Cl group was significantly lower than that in the pCDNA3.1 + NH_4_Cl group at 3, 24, and 48 h. The expression of the anti-inflammatory factor *IL-10* decreased significantly at 3 h after NH_4_Cl stimulation and reached the lowest level at 12 h. At the same time, the expression levels of oxidative stress-related genes (*GPX* and *SOD*) were transiently up-regulated at 3 h after NH_4_Cl stimulation and reached the lowest level at 12 h. At 48 h following NH_4_Cl stimulation, the expression levels of *GPX*, *SOD*, and *HO-1* in the pCDNA3.1 + NH_4_Cl group were markedly higher than those in the pCDNA3.1-On*TRX* + NH_4_Cl group (Figure 9D). Under NH_4_Cl stimulation, On*TRX* overexpression was also associated with higher transcript levels of *HSP70* and *P62*, two genes related to protein homeostasis and autophagy regulation. Specifically, the expression levels of *HSP70* and *P62* in the pCDNA3.1-On*TRX* + NH_4_Cl group reached their peaks at 48 h after stimulation (Figure 9A). These results indicate that the overexpression of On*TRX* is related to the expression changes of various stress-related genes.

### 3.8. Apoptosis Induced by NH_4_Cl

**Figure 10:**
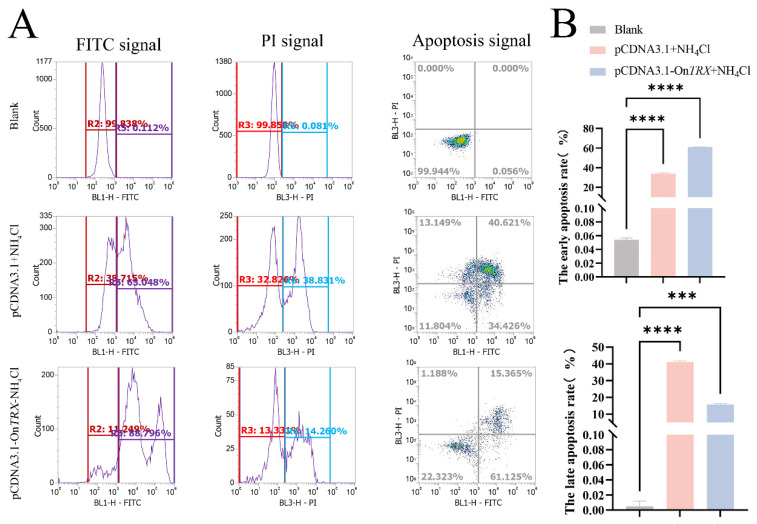
(**A**) Representative Annexin V-FITC/PI flow-cytometry plots showing apoptosis in the three TSE-04 groups (derived from Nile tilapia, *Oreochromis niloticus*). In the scatter plot, the yellower the color, the higher the cell density. (**B**) Quantitative analysis of apoptotic cells in each group. Data are presented as mean ± SD from three independent biological replicates. *** *p* < 0.001, **** *p* < 0.0001.

Flow cytometry was performed to evaluate whether On*TRX* overexpression could alleviate NH_4_Cl-induced apoptosis in TSE-04 cells (Figure 10A). In the control group, most cells remained viable, with minimal Annexin V-FITC or PI staining. After NH_4_Cl treatment, the proportion of apoptotic cells increased markedly, indicating that ammonia exposure induced substantial cell apoptosis. In contrast, the pCDNA3.1-On*TRX* + NH_4_Cl group showed a reduced apoptotic response compared with the pCDNA3.1 + NH_4_Cl group. Quantitative analysis demonstrated that On*TRX* overexpression significantly reduced the proportion of late apoptotic cells under NH_4_Cl exposure (Figure 10B), indicating that On*TRX* overexpression was associated with a lower proportion of late apoptotic cells under NH_4_Cl exposure.

## 4. Discussion

Oxidative stress originates from the imbalance between ROS production and antioxidant capacity in cells, and its direct consequence is oxidative damage of lipids, protein, and nucleic acids, which in turn triggers cell apoptosis or necrosis [25]. In intensive aquaculture, the increase in ammonia nitrogen concentration is the main environmental factor inducing oxidative stress in fish. Ammonia stress in fish has been reported to disrupt antioxidant enzyme systems, including SOD and CAT, and to reduce total antioxidant capacity (T-AOC) [26,27,28]. TRX, as a classical redox regulatory protein, plays a key role in alleviating oxidative stress and maintaining redox homeostasis in organisms [29]. The conserved active-site motif of TRX (Cys-Gly-Pro-Cys) participates in the reduction of oxidized proteins through the NADPH-dependent thioredoxin reductase (TRXR) system, thereby contributing to cellular redox homeostasis [30]. Under the condition of oxidative stress, the expression of *TRX* is significantly increased, which can reduce the level of ROS in cells, thus reducing membrane lipid peroxidation and DNA breakage [15]. However, at present, the research on the antioxidant function of On*TRX* in tilapia under ammonia exposure stress is still limited. In this study, the On*TRX* gene with an ORF of 324bp was successfully cloned, which encodes 107 amino acids and contains the thioredoxin domain. The results of multi-sequence alignment showed that On*TRX* is highly conserved in vertebrate fish. Phylogenetic tree analysis showed that On*TRX* of fish and mammals clustered on adjacent evolutionary branches. In contrast, the On*TRX* of arthropods formed an independent and distant evolutionary branch, which was significantly different from that of fish and mammals. These findings support the conserved nature of On*TRX* and provide a molecular basis for further investigation of its function in tilapia.

In fish, NH_4_Cl treatment and other exogenous stimuli can induce oxidative stress, and then affect the expression of *TRX* [19,31]. For example, in rainbow trout (*O. mykiss*), splenic *TRX* expression increased 2.4 fold 24 h after intraperitoneal injection of *Aeromonas hydrophila* [19]. In addition to pathogen challenge, members of the thioredoxin-related redox system in fish have also been reported to respond to environmentally relevant stressors associated with oxidative imbalance, such as fluctuations in temperature and salinity, as well as toxicant exposure [32,33]. During the 72 h ammonia exposure in this study, On*TRX* showed a significant induction pattern in all organs/tissues, but the peak time was obviously tissue specific: the intestinal tract reached its peak at 6 h after exposure, the highest expression in gill and liver appeared at 12 h, and the head kidney, skin, and spleen reached its peak at 24 h. The pronounced induction of On*TRX* in the gill is of particular interest because the gill is the primary interface between fish and the aquatic environment and therefore a major site of direct ammonia exposure. In addition to gas exchange, gills are essential for ion transport, acid–base balance, and nitrogenous waste exchange, making them especially sensitive to ammonia-induced oxidative stress [34]. By contrast, the liver is more closely associated with metabolic regulation and detoxification, and its altered On*TRX* expression may reflect a different aspect of the systemic response to ammonia challenge. Thus, the tissue-specific expression pattern of On*TRX* likely reflects the distinct physiological roles of individual organs during ammonia stress adaptation. These findings indicate that On*TRX* expression in tilapia is organ and tissue specific in response to ammonia exposure. However, the existing research on the influence of environmental factors on skin is still relatively scarce. As an organ in direct contact with the water environment, the main function of skin is to act as a barrier. Secondly, fish skin also plays an important role in the transportation of gases, ions, nitrogen-containing wastes, and nutrients [35,36]. In this study, the expression changes of On*TRX* in the skin epithelial cell line TSE-04 of Nile tilapia exposed to NH_4_Cl were detected. At 0,3,6, and 12 h after stimulation, there was no significant difference in the relative expression level of On*TRX*, and it reached the lowest value at 6 h; its expression began to increase gradually after 12 h, increased significantly at 24 h, and reached the peak at 48 h. This dynamic pattern suggests that On*TRX* may participate in the later transcriptional response of TSE-04 cells to NH_4_Cl-induced stress.

To further explore the potential role of On*TRX*, the recombinant plasmid pCDNA3.1-On*TRX* was constructed and overexpressed in TSE-04 cells. Ammonia exposure is known to exert toxic effects on aquatic organisms and cultured cells [37,38]. In the present study, On*TRX* overexpression was associated with reduced morphological damage in TSE-04 cells following NH_4_Cl exposure. In addition, qRT-PCR analysis showed that OnTRX overexpression was associated with altered expression of several stress-related genes, including *TNFα*, *IL10*, *GPX*, *SOD*, *HO-1*, *mTOR*, *HSP70*, and *P62*. These changes suggest that On*TRX* overexpression may influence the transcriptional response of TSE-04 cells under ammonia stress. Ammonia exposure stimulation promotes the production of *HSP70*, maintains the stability of cell structure, and reduces protein metabolism [39]. *TNFα* can cause inflammation and stimulate the immune system [40]. *TRX* has also been implicated in the redox control of inflammatory signaling, including *NF-κB*-related pathways. Because *NF-κB* activity is sensitive to intracellular redox status, changes in *TRX* abundance may influence the transcription of inflammatory mediators [41]. In the present study, the lower *TNFα* transcript levels observed in the On*TRX*-overexpression group may be compatible with a modulatory effect of On*TRX* on redox-sensitive inflammatory signaling; however, expression of *NF-κB* was not directly assessed. Further studies are still needed to validate the regulatory effect of On*TRX* on NF-κB in future investigations. When cells are stimulated by external sources, the *mTOR* signaling pathway is activated to promote anabolism and maintain cell survival [42]. *P62* (SQSTM1) is an autophagy receptor protein, which participates in the autophagy process, and can recognize and bind the damaged protein and organelles, and target them to autophagy for degradation [43]. When exposed to ammonia nitrogen, *P62* will be activated and regulate autophagy [44]. The higher transcript levels of *mTOR*, *P62*, and *HSP70* in On*TRX*-overexpressing cells suggest that On*TRX* modulates pathways involved in cell maintenance, protein homeostasis, and stress adaptation under ammonia exposure. The lower transcript levels of *GPX*, *SOD*, and *HO-1* in the On*TRX*-overexpression group relative to the ammonia-only group may reflect an attenuated cellular stress response; nevertheless, this interpretation remains speculative. Similarly, the elevated expression of *mTOR* and *P62* implies a potential link to stress adaptation, cellular maintenance, and damage response, though our current data cannot confirm direct activation of the *mTOR* pathway or changes in autophagic flux. Therefore, these transcript-level findings should be considered preliminary evidence supporting the involvement of On*TRX* in the cellular response to ammonia stress. Although direct protein-level validation was not performed in this study, the gene expression profiles strongly support the regulatory roles of On*TRX* in oxidative stress autophagy in tilapia. The potential broader functions of On*TRX*, including antioxidant, anti-apoptotic, and immunomodulatory effects, remain to be further verified at both the protein level and through gene knockdown assays in future work.

Flow cytometry results further showed that NH_4_Cl exposure had marked cytotoxic effects on TSE-04 cells. Compared with the blank control group, the pCDNA3.1 + NH_4_Cl group exhibited a substantial increase in both early and late apoptotic cells, whereas On*TRX* overexpression reduced the proportion of late apoptotic cells and increased the proportion of normal cells. These data support the conclusion that On*TRX* overexpression was associated with partial alleviation of NH_4_Cl-induced cellular injury in vitro. These results suggest that On*TRX* overexpression partially alleviated NH_4_Cl induced cellular injury in TSE-04 cells. Apoptosis may occur in some damaged cells under stress conditions before more severe injury develops [45,46]. This study was conducted in vitro (TSE-04 cells) and under acute ammonia exposure; future studies should explore the role of OnTRX in chronic ammonia stress and in vivo models. In mammals, *TRX* is known to bind apoptosis signal-regulating kinase 1 (ASK1) under reducing conditions, thereby suppressing ASK1 activation and downstream stress kinase signaling. Under oxidative stress, dissociation of *TRX* from ASK1 can promote apoptosis [47]. Although ASK1 was not examined in the present study, the reduced proportion of apoptotic cells in the On*TRX*-overexpression group is consistent with the possibility that On*TRX* may modulate ammonia-induced cell death through a conserved *TRX*-related anti-apoptotic mechanism.

## 5. Conclusions

In summary, the On*TRX* gene was successfully identified from Nile tilapia in this study, and its ORF length is 324 bp. On*TRX* was expressed in multiple tissues and showed significant transcriptional responses to ammonia exposure in the gill, head kidney, intestine, skin, liver, and spleen. In the TSE-04 skin epithelial cell line, On*TRX* overexpression alleviated ammonia-induced cellular injury, altered the expression of multiple stress-related genes, and reduced the proportion of late apoptotic cells. This study indicates that On*TRX* plays a crucial role in the defensive response of Nile tilapia to ammonia stress. Therefore, this study provides a useful basis for further exploring the mechanism of thioredoxin in the response of Nile tilapia to ammonia stress.

## Figures and Tables

**Table 1:** Primer sequences applied in the current study.

Gene Names	Sequence (5′–3′)
*TRX*-F *TRX*-R	CGGATCCGATGGTTTACGACGTGAAAGATCTGG CCTCGAGGTCATCTGAGCTCTCTCACTTTTTGA
Anti-pCDNA3.1-F Anti-pCDNA3.1-R	TCTAGAGGGCCCTACCCATACGATGT GGATCCGAGCTCGGTACCAAGCTTAAG
HA-*TRX*-F HA-*TRX*-R	TACCGAGCTCGGATCCCGGATCCGATGGTTTACGACGTGAAAGATCTGG GGTAGGGCCCTCTAGACCTCGAGGTCATCTGAGCTCTCTCACTTTTTGA
q*mTOR*-F q*mTOR*-R	TGTCCTCGCTCGTATTCC GGTCTTCTTCCTCCTCTGC
q*IL10*-F q*IL10*-R	GCTTCCCCGTCAGGCTCAA CTGTCGGCAGAACCGTGTC
q*GPX*-F q*GPX*-R	TTCATTCTCGCTACTCCG TCCATTCACATCCACCTT
q*TNFα*-F q*TNFα*-R	ATGTGAGAGCAGCCATTCAT ACAAAGTAGAGGCCATCTCG
q*HO-1*-F q*HO-1*-R	CAGATCGGCAGAGAGAACCC CTCTTTGCTGCTCAGACCGA
q*SOD*-F q*SOD*-R	GTGATCACCCTCACAGGTCC AGCATTACCGGTCTTCAGGC
q*Hsp70*-F q*Hsp70*-R	ATAAACCGCCAACTGTCC CCATCCTCCTCATTTCTTCT
q*p62*-F q*p62*-R	GCTAAGGGGAAGCACACTGA TTCAGGAAGTCCACGTTGGT
q*β-actin*-F q*β-actin*-R	AGATGAAATCGCCGCACTGG TCTGACCCATACCCACCATCA

## Data Availability

The data presented in this study are available from the corresponding author upon reasonable request.

## Abbreviations

The following abbreviations are used in this manuscript:

TRX	Thioredoxin
On-TRX	*Oreochromis niloticus* Thioredoxin
